# An Index of Treatment by Various Writers

**Published:** 1908-12

**Authors:** 


**An Index of Treatment by Various Writers.** Edited by Robert Hutchison, M.D., F.R.C.P., and H. Stansfield Collier, F.R.C.S. Pp. xiii., 877. Bristol: John Wright & Sons, Ltd. 1907.

There is no class of book whose value it is more difficult to correctly estimate than one arranged in the dictionary form, the subjects merely coming in alphabetical order. Presumably it is intended that the practitioner shall be able to take such a volume from his shelves when his own resources are exhausted, and rapidly imbibe from its pages new inspiration as to treatment. Looked at from this point of view, it is important that the recommendation should embrace every possible therapeutic device or agent which has proved efficacious even in hands other than those of the writer of the particular paragraph. It is true that for the most part this is fully carried into effect in the present work, and many excellent surveys of treatment are included, notably those on "Hæmorrhoids" and "Rheumatism." Dr. Bulloch's article on "Bacterio therapeutics," extending over twenty pages, contains, however, quite the most valuable information which the book conveys.

Elsewhere there is sometimes a tendency to insist on one line of treatment which may have proved successful in the writer's experience, to the exclusion of alternative suggestions. A bad example of this is to be found under the heading "Dyspepsia," where, if anywhere, there is scope for alternative suggestions. The subject is dismissed in a relatively small space, and contains nothing of interest or value to a harassed doctor at his wits' end for some really practical hints on this common and long-lasting complaint.

It would have been better, too, if in treating of "Inflammations of Bone," so-called Growth Fever had been relegated to a less prominent place. Unfortunately, the tendency to fall back on this or some equally slip-shod diagnosis is responsible for only too many delays in operating on the more common and more dangerous forms of acute panosteitis or epiphysitis.

Apart from a few blemishes of this nature, which indicate the erudition of the writer rather than judgment and practical experience, the book is a useful one; though it must be confessed that unless the publishers intend to bring out a revised edition every year, this *Index of Treatment* falls far short of the well-known *Wright's Annual*, which has the supreme advantage of being up to date, and scarcely less comprehensive in scope.

Perhaps the best advice we can give is that this volume should be first purchased and followed up by the successive volumes of the *Annual* as they appear.

